# Medication-assisted quit rates in participants with smoking-related diseases in EAGLES: Post hoc analyses of a double-blind, randomized, placebo-controlled clinical trial

**DOI:** 10.18332/tid/146567

**Published:** 2022-05-10

**Authors:** Philip Tønnesen, David Lawrence, Serena Tonstad

**Affiliations:** 1 Pulmonary Medicine, Søernes Privathospital, Frederiksberg, Denmark; 2 Center for Sleep Medicine, Søernes Privathospital, Frederiksberg, Denmark; 3 Global Product Development, Pfizer Inc, New York, United States; 4 Department of Preventive Cardiology, Oslo University Hospital, Oslo, Norway

## Abstract

**INTRODUCTION:**

Greater understanding is required of how smokers with smoking-related diseases respond to smoking cessation medications. This *post hoc* analysis of EAGLES data compared continuous abstinence rates (CARs) in smokers with/without smoking-related diseases and assessed participant demographic and baseline characteristics that may serve as predictors of continuous abstinence (CA).

**METHODS:**

EAGLES was a 24-week (12-week treatment, 12-week follow-up), double-blind, active- (nicotine replacement therapy; patch) and placebo-controlled study in motivated-to-quit smokers with/without psychiatric disorders. This analysis assessed CARs at weeks 9-12 (CAR9-12) and 9-24 (CAR9-24) in participants with smoking-related diseases [asthma, chronic obstructive pulmonary disease (COPD), diabetes, and/or cardiovascular disease (n=1372)] versus controls without these comorbidities (n=6039). Participants received varenicline 1 mg twice daily, bupropion 150 mg twice daily, nicotine patches 21 mg/day with taper, or placebo for 12 weeks. Stepwise logistic modeling was also performed to analyze odds ratio (OR) for predictors of CA at weeks 9-12 (CA9-12) and 9-24 (CA9-24).

**RESULTS:**

Smokers with smoking-related diseases were older, had a longer smoking history, more quit attempts, and were more likely to have a psychiatric disorder and reside in the US versus smokers without comorbidities. Fagerström Test for Cigarette Dependence scores and treatment adherence were comparable between cohorts. Smokers with smoking-related diseases had lower CARs versus controls (CAR9-12: 20.8% vs 24.0%; CAR9-24: 13.0% vs 16.9%). Use of smoking cessation medication was the strongest predictor of CA after control for demographics, smoking characteristics, and psychiatric disorder. By treatment, OR and CI were: varenicline CA9-12 (OR=3.82; 95% CI: 3.21-4.54) and CA9-24 (OR=2.92; 95% CI: 2.40-3.54); bupropion CA9-12 (OR=2.17; 95% CI: 1.81-2.60) and CA9-24 (OR=1.99; 95% CI: 1.63-2.44); nicotine patches CA9-12 (OR=2.23; 95% CI: 1.87-2.67) and CA9-24 (OR=1.86; 95% CI: 1.52-2.28).

**CONCLUSIONS:**

Smokers with smoking-related diseases had lower quit rates than controls. Of the active treatments compared, varenicline was most effective in smokers with asthma, COPD, diabetes, or cardiovascular disease.

**TRIAL REGISTRATION:**

NCT01456936 (https://clinicaltrials.gov/ct2/show/NCT01456936).

## INTRODUCTION

Globally, smoking is the second leading risk factor for deaths and disability-adjusted life-years after elevated systolic blood pressure^1^ and two-thirds of smokers die of a smoking-related disease^2^. Encouraging smokers to quit is critically important^3^, as smoking cessation is considered an effective intervention by which to lower the risk of death and disability associated with a range of smoking-related chronic diseases^4^.

Lung diseases are a major consequence of cigarette smoking^4^. Studies in smokers with chronic obstructive pulmonary disease (COPD) have demonstrated the effectiveness of behavioral treatment and pharmacotherapy in aiding smoking cessation^5^. However, up to 40% of smokers with COPD may continue to smoke, possibly due to a high degree of nicotine dependence^6^. Studies have found smoking rates in patients with asthma to be similar to those in the general population^7^. Control of asthma symptoms and bronchial reactivity tends to improve substantially in smokers with asthma who quit, while inhaled anti-asthmatic medication is more efficacious in non-smokers than in those who smoke^6^. Despite these potential benefits, smoking cessation trials specifically tailored to patients with asthma are lacking^6^.

Smokers with cardiovascular disease (CVD) are most likely to quit following an acute event compared with smokers with other smoking-related diseases^8^. In the acute CVD setting and in smokers with stable CVD, smoking cessation pharmacotherapies (in particular, bupropion and varenicline) are shown to be effective^9^. However, psychosocial and other triggers appear to contribute to the resumption of smoking in up to 50% of quitters as the acute phase of the illness abates^10,11^.

Reported rates of smoking vary among patients with type 2 diabetes^8,12^ and studies of the effectiveness of smoking cessation medications are limited. Smoking contributes to abnormal fat distribution, adversely affects insulin sensitivity, and reduces glucose disposal^13^; however, cessation may be hindered by fear of weight gain and loss of glycemic control. Although some studies show worsening of diabetes post cessation, this phase seems to normalize with longer follow-up^14,15^. Furthermore, weight gain after smoking cessation does not negate the beneficial cardiovascular effects of quitting^15,16^.

To help increase quit rates in smokers with smoking-related diseases, greater understanding is needed of whether these smokers differ in their smoking characteristics compared with smokers without comorbid conditions, and whether the response to smoking cessation pharmacotherapies and behavioral support differs between these populations.

EAGLES (Evaluating Adverse Events in a Global Smoking Cessation Study) was a large (n=8144), phase IV, smoking cessation, randomized clinical trial (RCT) that captured detailed individual participant data with regard to smoking-related diseases^17^. The objectives of this *post hoc* analysis of EAGLES efficacy and safety data were to compare carbon monoxide (CO)-confirmed continuous abstinence rates (CARs) in smokers with asthma, COPD, diabetes, and/or CVD versus smokers without these comorbidities, and to assess participant demographic and baseline characteristics that may serve as predictors of continuous abstinence (CA). A plain language summary is available for this article in Supplementary Material.

## METHODS

### Study design and population

EAGLES (ClinicalTrials.gov: NCT01456936) was a 24-week (12-week treatment, 12-week non-treatment follow-up), double-blind, active- [nicotine replacement therapy (NRT): nicotine patch 21 mg/day with taper] and placebo-controlled, multicenter, parallel-group, triple-dummy RCT designed to assess the safety and efficacy of varenicline 1 mg twice daily and bupropion 150 mg twice daily for smoking cessation in smokers with and without pre-specified psychiatric diagnoses.

A detailed description of the EAGLES design, population, treatment regimens, and outcome measures was published previously^17^. Briefly, the study was conducted between November 2011 and January 2015 at 140 centers in the US and 15 other countries. Participants were male and female smokers, aged 18-75 years, who smoked an average of ≥10 cigarettes per day and who were motivated to stop smoking. Inclusion criteria for the psychiatric cohort required an established and stable diagnosis of psychiatric disorder conducted at screening and confirmed by the Structured Clinical Interview for the Diagnostic and Statistical Manual of Mental Disorders 4th edition Axis I and II disorders^18,19^. The primary neuropsychiatric adverse event (NPSAE) endpoint was a novel composite of 16 components, which comprised 261 pre-specified Medical Dictionary for Regulatory Activities preferred terms. Efficacy endpoints compared CO-confirmed CARs for smokers treated with active treatments and placebo at weeks 9-12 (CAR9-12) and weeks 9-24 (CAR9-24).

For this *post hoc* analysis, EAGLES participants were stratified into the smoking-related-disease cohort (those with a self-reported history of asthma, COPD, diabetes, or CVD), or the control cohort (without these comorbidities) (Supplementary file Figure S1). Because of the heterogeneity of cancer diagnoses, smokers who reported present, active cancer (n=2: one diagnosis each of colon cancer and non-Hodgkin lymphoma) were excluded. Likewise, since alcoholism may confound the relationship between smoking and smoking-related diseases and possibly lower overall adherence, those who reported past or present alcohol dependence were also excluded. Diagnoses included alcohol abuse (n=553), alcohol dependence syndrome (n=584), and alcoholism (n=64); participants may have one or more diagnosis (cumulative frequency: n=715). Participants with past or present schizophrenia (n=18) were excluded because of possible bias in the analysis due to poor medicine compliance or co-abuse of other substances that can contribute to lower quit rates in this population.

### Statistical analysis

Demographic and other baseline characteristics were summarized by cohort and by smoking-related-disease subcohort; participants with more than one smoking-related disease could be assigned to multiple subcohorts. CO-confirmed CAR9-12 and CAR9-24 were presented by: 1) cohort and by smoking-related-disease subcohort; and 2) treatment and cohort/smoking-related-disease subcohort. The incidence of NPSAEs and study-drug exposure (days) were summarized by treatment and cohort/smoking-related-disease subcohort. Common adverse events (AEs) (≥5% of participants in any treatment by cohort) and change in body weight from baseline were summarized by treatment and cohort. Descriptive statistics included the number of observations, percentage, mean, and standard deviation (SD).

Stepwise logistic regression was performed to assess predictors of CA for weeks 9-12 (CA9-12) and weeks 9-24 (CA9-24). The model forced inclusion of treatment (varenicline, bupropion, NRT, or placebo), psychiatric cohort (psychiatric vs non-psychiatric), medical history cohort (smoking-related disease vs control), and region (non-USA vs USA) terms. Demographic and other baseline characteristic variables were considered as candidate terms, as was a term for treatment by medical history cohort interaction. Analyses were not pre-planned and should therefore be considered exploratory.

## RESULTS

### Study population

The mean (SD) age was 52.2 (11.8) years in smokers with smoking-related diseases (n=1372) versus 45.1 (12.2) years in controls (n=6039); the respective mean (SD) number of years smoked was 33.8 (12.5) versus 26.9 (12.0) years (Table 1). Baseline history of psychiatric disease was recorded in 705 (51.4%) smokers with smoking-related diseases versus 2693 (44.6%) in controls; the number of smokers who resided in the US was 745 (54.3%) versus 2974 (49.2%), respectively. Prior use of varenicline, bupropion, and NRT, and the number of previous quit attempts, was numerically higher in each of the smoking-related-disease subcohorts compared with controls. Fagerström Test for Cigarette Dependence (FTCD) mean (SD) score and baseline CO were the same in the smoking-related-disease cohort and controls [FTCD: 5.7 (2.0); CO: 21 ppm] and mean (SD) daily cigarette use over the previous month was also similar [21.3 (8.9) vs 20.5 (8.0)]. Mean (SD) body mass index was 29.5 (6.7) versus 27.7 (6.2) kg/m^2^ in the smoking-related-disease cohort versus controls.

**Table 1:** Characteristics of all randomized participants in EAGLES by cohort and by smoking-related-disease subcohort

	*Asthma (n=486)*	*COPD (n=412)*	*Diabetes (n=409)*	*CVD (n=285)*	*All disease subcohorts ^a^ (n=1372)*	*No smoking-related diseases ^b^ (n=6039)*
**Demographic characteristics^c^**						
**Male**, n (%)	148 (30.5)	166 (40.3)	191 (46.7)	178 (62.5)	593 (43.2)	2612 (43.3)
**Age** (years), mean ± SD	45.0 ± 12.3	56.9 ± 9.0	54.8 ± 9.4	59.1 ± 8.5	52.2 ± 11.8	45.1 ± 12.2
**Race**, n (%)						
White	366 (75.3)	350 (85.0)	293 (71.6)	245 (86.0)	1084 (79.0)	5005 (82.9)
Black	91 (18.7)	51 (12.4)	92 (22.5)	32 (11.2)	223 (16.3)	807 (13.4)
Other	29 (6.0)	11 (2.7)	24 (5.9)	8 (2.8)	65 (4.7)	227 (3.8)
**BMI** (kg/m^2^), mean ± SD	29.8 ± 6.8	28.0 ± 6.6	32.1 ± 6.7	28.3 ± 5.5	29.5 ± 6.7	27.7 ± 6.2
**Region**, n (%)						
USA	294 (60.5)	200 (48.5)	237 (57.9)	157 (55.1)	745 (54.3)	2974 (49.2)
Non-USA	192 (39.5)	212 (51.5)	172 (42.1)	128 (44.9)	627 (45.7)	3065 (50.8)
**Smoking characteristics**						
FTCD score, mean ± SD	5.7 ± 2.0	6.0 ± 1.8	5.7 ± 2.0	5.6 ± 1.9	5.7 ± 2.0	5.7 ± 2.0
Baseline CO (ppm), mean ± SD	20 ± 10	22 ± 10	22 ± 11	22 ± 10	21 ± 10	21 ± 10
Years smoked, mean ± SD	26.6 ± 12.2	39.2 ± 9.8	35.5 ± 11.2	40.5 ± 10.5	33.8 ± 12.5	26.9 ± 12.0
Cigarettes smoked per day in past month, mean ± SD	20.2 ± 8.5	22.9 ± 9.8	21.6 ± 9.7	21.5 ± 8.5	21.3 ± 8.9	20.5 ± 8.0
Lifetime quit attempts, n (%)	422 (86.8)	352 (85.4)	349 (85.3)	253 (88.8)	1174 (85.6)	4890 (81.0)
Number of lifetime serious^d^ quit attempts, mean ± SD	4.6 ± 19.2	3.8 ± 5.3	3.6 ± 5.9	3.6 ± 3.9	4.0 ± 12.2	3.0 ± 7.6
Living with a smoker, n (%)	189 (38.9)	144 (35.0)	139 (34.0)	85 (29.8)	474 (34.5)	2188 (36.2)
Contact with a smoker, n (%)	349 (71.8)	278 (67.5)	267 (65.3)	168 (58.9)	912 (66.5)	4259 (70.5)
**Prior use of study treatments**						
Varenicline, n (%)	103 (21.2)	103 (25.0)	94 (23.0)	83 (29.1)	320 (23.3)	781 (12.9)
Bupropion, n (%)	69 (14.2)	65 (15.8)	62 (15.2)	48 (16.8)	197 (14.4)	531 (8.8)
NRT, n (%)	148 (30.5)	139 (33.7)	131 (32.0)	114 (40.0)	437 (31.9)	1397 (23.1)
**Psychiatric history**						
Psychiatric cohort, n (%)	264 (54.3)	212 (51.5)	222 (54.3)	123 (43.2)	705 (51.4)	2693 (44.6)
Non-psychiatric cohort, n (%)	222 (45.7)	200 (48.5)	187 (45.7)	162 (56.8)	667 (48.6)	3346 (55.4)
Lifetime C-SSRS ideation, n (%)	113 (23.3)	83 (20.1)	82 (20.0)	43 (15.1)	271 (19.8)	945 (15.6)
Lifetime C-SSRS behavior, n (%)	49 (10.1)	34 (8.3)	29 (7.1)	21 (7.4)	111 (8.1)	277 (4.6)
HADS Anxiety subscale score, mean ± SD	4.5 ± 3.7	4.7 ± 3.7	4.5 ± 3.8	4.0 ± 3.5	4.4 ± 3.7	3.7 ± 3.4
HADS Depression subscale score, mean ± SD	2.5 ± 2.9	3.3 ± 3.3	3.1 ± 3.2	2.7 ± 2.9	2.8 ± 3.1	2.2 ± 2.7

a Participants may appear in multiple smoking-related-disease subcohorts.

b Participants without asthma, COPD, diabetes, or CVD.

c Baseline missingness was negligible: BMI had 0.6% missingness, with other variables generally 0–0.1%.

d ≥24 hours. BMI: body mass index.

CO: carbon monoxide. COPD: chronic obstructive pulmonary disease. C-SSRS: Columbia-Suicide Severity Rating Scale. CVD: cardiovascular disease. FTCD: Fagerström Test for Cigarette Dependence. HADS: Hospital Anxiety and Depression Scale. NRT: nicotine replacement therapy. ppm: parts per million. SD: standard deviation.

### Continuous abstinence rates

**Figure 1:**
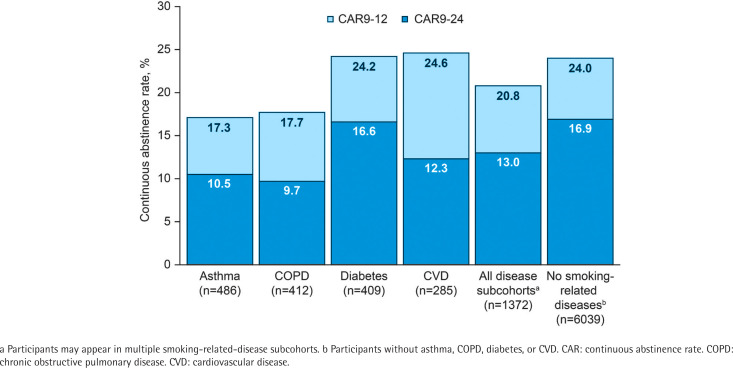
Continuous abstinence rates for weeks 9-12 (CAR9-12) and 9-24 (CAR9-24) by cohort and smoking-related-disease subcohort in all randomized EAGLES participants

CAR9-12 and CAR9-24 were 285/1372 (20.8%) versus 1450/6039 (24.0%) in the overall smoking-related-disease cohort and 179/1372 (13.0%) versus 1019/6039 (16.9%) in controls (Figure 1). CAR9-12 was 99/409 (24.2%) in smokers with diabetes and 70/285 (24.6%) in smokers with CVD, compared with patients with asthma [84/486 (17.3%)] and COPD [73/412 (17.7%)] (Figure 1). CAR9-24 was 68/409 (16.6%) in smokers with diabetes, whereas CAR9-24 was 51/486 (10.5%), 40/412 (9.7%), and 35/285 (12.3%), for smokers with asthma, COPD, and CVD, respectively (Figure 1).

Results were further stratified by active treatment (varenicline, bupropion, NRT) or placebo (Supplementary file Table S1). For smokers with asthma or COPD, CAR9-12 and CAR9-24 were lower with all active treatments compared with controls. Conversely, for smokers with diabetes, CAR9-12 and CAR9-24 were similar or higher compared with the control cohort. For smokers with CVD, CAR9-12 was similar to the control cohort for all active treatments; however, CAR9-24 was lower compared with controls. In the smoking-related-disease and control cohorts CAR9-12 and CAR9-24 were higher in smokers treated with varenicline compared with bupropion or NRT. Following treatment with placebo, markedly lower CAR9-12 and CAR9-24 were observed for smokers with COPD or diabetes compared with the control group; however, quit rates were similar in smokers with asthma or CVD compared with controls.

### Predictors of abstinence

**Figure 2:**
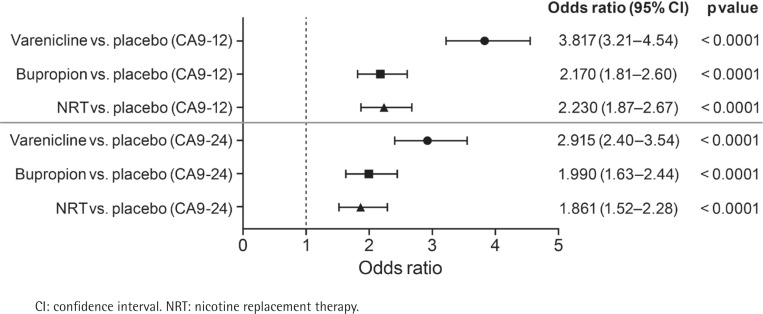
Stepwise logistic modeling: use of smoking cessation medication as a predictor of continuous abstinence at weeks 9-12 (CA9-12) and 9-24 (CA9-24) in all randomized EAGLES participants

For all randomized participants, a stepwise, logistic modeling analysis of predictors of CA9-12 and CA9-24 was conducted. Use of a smoking cessation medication was a significant predictor of CA at weeks 9-12: varenicline (OR=3.817; 95% CI: 3.21-4.54); bupropion (OR=2.170; 95% CI: 1.81-2.60); NRT (OR=2.230; 95% CI: 1.87-2.67) (Figure 2). Other significant positive predictors of CA included residing outside the US, older age, and previous attempt to quit (Supplementary file Table S2). Comorbid smoking-related disease or psychiatric diagnosis were inversely associated with CA: OR=0.797 (95% CI: 0.68-0.93) and OR=0.742 (95% CI: 0.66-0.83), respectively. FTCD score, Black race, daily/regular contact with a smoker, and cigarettes per day smoked in the past month were also inversely associated with CA at weeks 9-12 (Supplementary file Table S2).

Predictors of CA at weeks 9-12 were generally also predictors of CA at weeks 9-24; however, ORs for CA with varenicline, bupropion, and NRT treatment were lower at weeks 9-24 (Table 2). Comorbid smoking-related disease (OR=0.705; 95% CI: 0.59-0.85) and psychiatric diagnosis (OR=0.720; 95% CI: 0.63-0.82) were inversely associated with CA at weeks 9-24. Previous attempts to quit did not predict CA at weeks 9-24; however, higher body mass index was predictive of CA over this follow-up period.

**Table 2:** Occurrence and incidence of neuropsychiatric adverse events and study drug adherence, by treatment and cohort, and by smoking-related-disease subcohort: all treated EAGLES participants

*Treatments*	*Asthma*	*COPD*	*Diabetes*	*CVD*	*All disease subcohorts ^a^*	*No smoking-related diseases ^b^*
**Neuropsychiatric adverse events**, N*, n (%)						
All treatments	481	411	408	283	1363	5970
	19 (4.0)	16 (3.9)	17 (4.2)	7 (2.5)	48 (3.5)	208 (3.5)
Varenicline	129	98	100	73	340	1505
	2 (1.6)	2 (2.0)	4 (4.0)	1 (1.4)	9 (2.6)	53 (3.5)
Bupropion	101	98	110	73	333	1494
	6 (5.9)	7 (7.1)	4 (3.6)	3 (4.1)	16 (4.8)	55 (3.7)
NRT	123	98	95	74	336	1503
	8 (6.5)	5 (5.1)	8 (8.4)	2 (2.7)	16 (4.8)	47 (3.1)
Placebo	128	117	103	63	354	1468
	3 (2.3)	2 (1.7)	1 (1.0)	1 (1.6)	7 (2.0)	53 (3.6)
**Study drug exposure**, N*, mean days (SD)						
All treatments	481	411	408	283	1363	5970
	72.3 (24.0)	72.5 (24.4)	75.2 (22.4)	73.4 (24.8)	73.0 (24.1)	74.4 (23.1)
Varenicline	129	98	100	73	340	1505
	71.6 (24.5)	71.3 (26.4)	76.4 (20.4)	75.2 (23.1)	72.7 (24.3)	75.1 (22.6)
Bupropion	101	98	110	73	333	1494
	74.4 (21.7)	74.2 (23.5)	72.3 (24.9)	75.4 (22.7)	72.9 (24.2)	74.3 (23.3)
NRT	123	98	95	74	336	1503
	71.9 (24.5)	73.0 (24.0)	77.1 (21.1)	72.2 (26.1)	74.0 (23.5)	73.8 (23.4)
Placebo	128	117	103	63	354	1468
	71.8 (25.0)	71.8 (23.9)	75.4 (22.6)	70.3 (27.5)	72.4 (24.6)	74.3 (23.2)

* N: population.

a Participants may appear in multiple smoking-related-disease subcohorts.

b Participants without asthma, COPD, diabetes, or CVD.

COPD: chronic obstructive pulmonary disease. CVD: cardiovascular disease. NRT: nicotine replacement therapy. SD: standard deviation.

Exploratory logistic regression analyses were conducted to explore the effect of each medication versus placebo across each smoking-related-disease subcohort. For CA at both weeks 9-12 and 9-24, results showed varenicline to be superior to placebo in the COPD [OR=4.42 (95% CI: 2.01-9.70) and OR=3.87 (95% CI: 1.42-10.58)] and diabetes subcohorts [OR=8.09 (95% CI: 3.49-18.76) and OR=6.87 (95% CI: 2.48-19.05)] and in controls [OR=3.74 (95% CI: 3.10-4.50) and OR=2.83 (95% CI: 2.29-3.48)], but not in the asthma or CVD subcohorts (Supplementary file Table S3). Similar results were observed for bupropion and NRT versus placebo at weeks 9-12; however, at weeks 9-24, superiority versus placebo was observed only in the diabetes subcohort and in controls (Supplementary file Table S3).

### Incidence of neuropsychiatric adverse events

The overall incidence of NPSAEs was 48/1363 (3.5%) for smokers in the smoking-related-disease cohort and 208/5970 (3.5%) for controls (Table 2). Regardless of treatment, the incidence of NPSAEs in smokers with CVD was 7/283 (2.5%) versus 19/481 (4.0%), 16/411 (3.9%), and 17/408 (4.2%) in those with asthma, COPD, or diabetes, respectively. For smokers who received varenicline, the incidence of NSPAEs was 9/340 (2.6%) in the smoking-related-disease cohort compared with 53/1505 (3.5%) in controls. In smokers with smoking-related disease, NPSAEs were reported in 16/333 (4.8%) who received bupropion and 16/336 (4.8%) receiving NRT versus 55/1494 (3.7%) and 47/1503 (3.1%) in controls, respectively (Table 2).

### Treatment adherence

Overall, the mean number of days (SD) for which smokers in the smoking-related-disease cohort adhered to their assigned study medication were 73.0 (24.1) versus 74.4 (23.1) for controls (Table 2). Regardless of treatment, mean adherence between smoking-related-disease subcohorts ranged between 72.3 and 75.2 days. Mean adherence was also similar between treatment groups overall, and when stratified by smoking-related-disease subcohort (Table 2).

### Weight change and adverse events

Mean change in body weight (kg) (SD) from baseline at week 12, independent of treatment, was 0.9 (2.9) for the smoking-related-disease cohort (n=1145) versus 1.0 (2.7) for controls (n=5068). At week 24, the mean change in body weight (kg) (SD) from baseline was 1.2 (3.9) versus 1.4 (3.7) for the smoking-related-disease cohort (n=1033) and controls (n=4516), respectively (Supplementary file Table S4). Weight gain was similar between the smoking-related-disease subcohorts.

Treatment-emergent AEs (reported in ≥5% of participants in any treatment) summarized by treatment and cohort are presented in Supplementary file Table S5. Overall, commonly reported AEs for the smoking-related-disease cohort (n=1363) versus controls (n=5970) were: nausea [186 (13.6%) vs 761 (12.7%)], headache [154 (11.3%) vs 635 (10.6%)], insomnia [136 (10.0%) vs 556 (9.3%)], abnormal dreams [120 (8.8%) vs 458 (7.7%)], nasopharyngitis [112 (8.2%) vs 431 (7.2%)], anxiety [102 (7.5%) vs 375 (6.3%)], upper respiratory tract infection [98 (7.2%) vs 266 (4.5%)], fatigue [76 (5.6%) vs 210 (3.5%)], dizziness [71 (5.2%) vs 230 (3.9%)], dry mouth [69 (5.1%) vs 237 (4.0%)], and irritability [59 (4.3%) vs 260 (4.4%)].

## DISCUSSION

Smokers in EAGLES who had already developed a smoking-related disease, including asthma, COPD, diabetes, or CVD, had lower quit rates than smokers without these comorbid conditions. However, the efficacy of smoking cessation medications generally remained higher than placebo, and all three medications that were tested in the trial showed significant benefits at the end of treatment (week 12) that persisted up to the end of follow-up at week 24. Varenicline showed the greatest effect, with non-overlapping CIs at both 12- and 24-week time points between varenicline and bupropion or NRT. Given that EAGLES is the largest medication-assisted smoking cessation trial to date, these results may be important to clinical practice.

When summarized by smoking-related diseases, results suggest the lower quit rates in the smoking-related disease cohort to be applicable to asthma, COPD, and (in a lesser sense) CVD only; smokers with diabetes had similar CARs at weeks 9-12 and 9-24 compared with controls receiving active treatment, but markedly lower quit rates with placebo.

Given the burden of smoking-related comorbidities, greater understanding is required of how smokers with smoking-related diseases respond to smoking cessation medications. Notably, they (overall, and in each smoking-related-disease subcohort) reported a higher number of previous serious quit attempts compared with controls, and a higher percentage prior use of varenicline, bupropion, and NRT. This suggests that lower quit rates in smokers with comorbidities are not due to an inherent lack of motivation to attempt to quit, nor a lack of willingness to use smoking cessation aids to do so. The study found varenicline was the most efficacious treatment, compared with bupropion and NRT, in both smokers with smoking-related diseases and controls, and aligns with recent tobacco treatment guidelines which recommend treatment initiation with varenicline over bupropion or NRT^20^. It is noted that the estimation-oriented approach for treatment comparison by smokingrelated disease subcohort is suggestive in nature due to the lower subcohort sample sizes and resulting wider confidence intervals.

We observed consistent mean study medication adherence rates among all smoking-related-disease subcohorts and controls (73 and 74 days out of 90 days, respectively). Our results suggest that neuropsychiatric safety is not a major concern when prescribing varenicline, bupropion, or NRT, to smokers with smoking-related diseases, and that commonly reported AEs were similar between smoking-related disease and control cohorts. Although the prospect of weight gain can be a concern to smokers wishing to quit, our results suggest no effect by cohort (smoking-related disease vs control) or treatment. However, as the most effective drug seems to be varenicline, our results suggest that this should be the drug of choice.

Overall, the presence of a comorbid smoking-related disease was identified as an independent predictor of significantly lower CA compared with controls at weeks 9-12 and 9-24, as was a psychiatric disease diagnosis. Smokers with smoking-related diseases were older and more likely to reside in the US compared with controls. Consistent with previous *post hoc* analyses of EAGLES data, older age was identified as a positive predictor of abstinence at weeks 9-12 and 9-24, whereas residing in the US was inversely associated with abstinence^21^. However, taken together, these differences do not explain the lower CARs in smokers with smoking-related diseases observed in the current analyses. Use of smoking cessation medication was by far the strongest positive predictor of abstinence.

Published data investigating quit rates in smokers with smoking-related diseases following treatment with smoking cessation pharmacotherapy are limited, but generally support the current analyses. In an RCT of 52 smokers with asthma who received varenicline or placebo, CARs at week 12 were significantly higher in the varenicline-treated group (69% vs 36%, respectively)^22^. However, between-treatment differences at 24 weeks were not significant due to a high relapse rate. In a recent open-label study^23^, 101 smokers who were hospitalized due to COPD, asthma, or pneumonia, self-selected to receive either varenicline plus intensive behavioral support versus no medicine and one consultation. Quit rates after 12 weeks were 55% versus 16% and after 1 year 52% versus 14% for the respective treatment regimens. A Cochrane meta-analysis that included 16 studies^5^ found that varenicline, bupropion, and NRT, were all effective treatments in smokers with COPD; however, the analysis did not compare quit rates in smokers without COPD. In the current study, nicotine dependence did not differ between the overall smoking-related disease cohort and controls; however, smokers with COPD had slightly higher FTCD scores versus controls (6.0 vs 5.7), and a higher mean daily cigarette consumption (22.9 vs 20.5).

A retrospective pooled analysis of 15 Pfizer-sponsored studies in which smokers were treated with varenicline, reported that those with diabetes (n=323) had higher CAR9-12 (43.8%), CAR9-24 (27.5%), and CAR9-52 (18.4%) compared with patients who received placebo^24^. However, in contrast to the current analyses, CARs at each endpoint were lower compared with smokers without diabetes. In addition, observed quit rates with placebo were higher than those in the current analysis (CAR9-12: 24.8%; CAR9-24: 14.4%). Since these studies were not designed explicitly to explore quit rates in smokers with diabetes, an RCT is warranted to confirm whether quit rates in smokers with diabetes are indeed similar to those without comorbid conditions.

In an RCT of smokers hospitalized with acute coronary syndrome (n=302) CAR9-24 was higher following 12 weeks of treatment with varenicline versus placebo^25^. However, studies in which patients with acute coronary syndrome were treated with bupropion or NRT were inconclusive or failed to show improvement versus placebo, particularly in the longer term^26,27^. In an RCT of smokers with stable CVD (n=714), CAR9-12 and CAR9-52 were significantly higher following treatment with varenicline versus placebo^28^. In smokers with acute myocardial infarction (n=392) randomized to receive bupropion versus placebo for 9 weeks, CARs at week 52 were not significantly different between treatments (26.8% vs 22.2%)^29^. A recent meta-analysis of RCTs investigating the efficacy of smoking cessation interventions in smokers with CVD found evidence relating to efficacy of NRT to be inconclusive^9^.

Notably, comparative smoking cessation trials of smokers with smoking-related diseases versus otherwise healthy smokers are lacking. We compared the quit rates observed for smokers with asthma, COPD, diabetes, and CVD, in the current analyses with those from RCTs conducted by Pfizer in which patients with the above-mentioned smoking-related diseases were enrolled^28,30^ and with studies comprising a general population of smokers^17,31,32^ (Supplementary file Figures S2 and S3). All studies included varenicline and placebo and were similarly designed. Taken together, the data support the findings presented in the current study: that quit rates are generally lower for patients with asthma, COPD, and CVD. The implications of lower quit rates in smokers with these smoking-related diseases — but not for smokers with diabetes — suggest that intensification of smoking cessation interventions for these populations of smokers may be beneficial. For example, regular re-treatment of smokers who relapse was described previously in a study of smokers with mild COPD who were referred for smoking cessation treatment every 4 months during a 5-year period^33^.

### Limitations

Our study has several limitations. First, EAGLES was an RCT and selection bias may have occurred because participants were a self-referred, motivated-to-quit population and not fully representative of the general population of smokers. Second, there may have been misclassification of participants to the smoking-related-disease subcohorts as medical diagnoses were self-reported and were not validated by medical records; therefore, the severity and the duration of these diseases were also not available. Third, although the overall smoking-related-disease cohort was relatively large (n=1372), the current analyses were not pre-planned and EAGLES was not powered to detect differences in quit rates in small subpopulations of smokers such as those stratified by smoking-related disease type.

## CONCLUSIONS

In this *post hoc* analysis of EAGLES data, we report that smokers with asthma, COPD, and CVD, have lower CARs compared with otherwise healthy smokers, despite a similar degree of nicotine dependence and similar treatment adherence. Smokers with diabetes had a similar quit rate compared with controls when taking active medication, and further studies are required to confirm these findings. Smokers with asthma, COPD, and CVD, may therefore benefit from increased behavioral and pharmacologic support for smoking cessation, which may include re-treatment following a failed quit attempt(s) or relapse.

## Data Availability

Upon request, and subject to review, Pfizer will provide the data that support the findings of this study. Subject to certain criteria, conditions and exceptions, Pfizer may also provide access to the related individual de-identified participant data. See https://www.pfizer.com/science/clinical-trials/trial-data-and-results for more information.
