# Supplementary material for: Medication-assisted quit rates in participants with smoking-related diseases in EAGLES: Post hoc analyses of a double-blind, randomized, placebo-controlled clinical trial
Source: Tob Induc Dis. 2022 May 10;20:46. doi: 10.18332/tid/146567 (PMC9086158; doi:10.18332/tid/146567)
Supplement: Supplementary file 1 [file TID-20-46-s1.pdf]

SUPPLEMENTARY MATERIAL

Table S1 Continuous abstinence rates for weeks 9–12 (CAR9-12) and 9–24 (CAR9-24) by treatment and cohort, and by smoking-related-disease subcohort in all randomized EAGLES participants[a]

	Asthma (n=486)	COPD (n=412)	Diabetes (n=409)	CVD (n=285)	All disease subcohorts[a] (n=1372)	No smoking-related diseases[b] (n=6039)
**Varenicline, n (%)**	**(n=131)**	**(n=99)**	**(n=100)**	**(n=74)**	**(n=344)**	**(n=1521)**
CAR9-12	29 (22.1)	27 (27.3)	39 (39.0)	21 (28.4)	103 (29.9)	530 (34.8)
CAR9-24	18 (13.7)	15 (15.2)	25 (25.0)	11 (14.9)	63 (18.3)	355 (23.3)
**Bupropion, n (%)**	**(n=103)**	**(n=98)**	**(n=110)**	**(n=74)**	**(n=336)**	**(n=1514)**
CAR9-12	18 (17.5)	17 (17.3)	26 (23.6)	20 (27.0)	70 (20.8)	356 (23.5)
CAR9-24	14 (13.6)	8 (8.2)	18 (16.4)	10 (13.5)	46 (13.7)	262 (17.3)
**NRT, n (%)**	**(n=124)**	**(n=98)**	**(n=96)**	**(n=74)**	**(n=338)**	**(n=1516)**
CAR9-12	16 (12.9)	18 (18.4)	26 (27.1)	20 (27.0)	69 (20.4)	372 (24.5)
CAR9-24	8 (6.5)	11 (11.2)	20 (20.8)	7 (9.5)	42 (12.4)	254 (16.8)
**Placebo, n (%)**	**(n=128)**	**(n=117)**	**(n=103)**	**(n=63)**	**(n=354)**	**(n=1488)**
CAR9-12	21 (16.4)	11 (9.4)	8 (7.8)	9 (14.3)	43 (12.1)	192 (12.9)
CAR9-24	11 (8.6)	6 (5.1)	5 (4.9)	7 (11.1)	28 (7.9)	148 (9.9)

[a]Participants may appear in multiple smoking-related-disease subcohorts.

[b]Participants without asthma, COPD, diabetes, or CVD.

CAR: continuous abstinence rate; COPD: chronic obstructive pulmonary disease; CVD: cardiovascular disease; NRT: nicotine replacement therapy.

Table S2 Stepwise logistic modeling: analysis of predictors of continuous abstinence for weeks 9–12 (CA9-12) and 9–24 (CA9-24) in all randomized EAGLES participants

		CA9-12			CA9-24		
**Model terms**	**Forced inclusion[a]**	**OR**	**95% CI**	**p**	**OR**	**95% CI**	**p**
Treatment[a]	Yes						
Smoking-related-disease cohort vs. controls	Yes	0.797	0.68–0.93	0.0042	0.705	0.59–0.85	0.0002
Psychiatric vs. non-psychiatric cohort	Yes	0.742	0.66–0.83	<0.0001	0.720	0.63–0.82	<0.0001
Region: non-USA vs. USA	Yes	1.984	1.76–2.24	<0.0001	1.997	1.73–2.31	<0.0001
Race	No						
Black vs. White		0.576	0.47–0.71	<0.0001	0.548	0.43–0.70	<0.0001
Other vs. White		0.779	0.57–1.06	0.1115	0.797	0.56–1.13	0.2028
Prior use of NRT (yes vs. no)	No	–	–	NS	0.783	0.67–0.92	0.0031
Contact with a smoker (yes vs. no)	No	0.784	0.69–0.89	0.0001	0.802	0.70–0.92	0.0020
Lifetime prior quit attempt (yes vs. no)	No	1.227	1.05–1.43	0.0097	–	–	NS
FTCD score (1-unit increase: higher score = higher dependence	No	0.918	0.89–0.95	<0.0001	0.925	0.89–0.96	<0.0001
Age, years (1-year increase)	No	1.012	1.01–1.02	<0.0001	1.013	1.01–1.02	<0.0001
BMI, kg/m^2 (1-unit increase)	No	–	–	NS	1.017	1.01–1.03	0.0028
Cigarettes per day in the past month (1-cigarette increase)	No	0.971	0.96–0.98	<0.0001	0.973	0.96–0.98	<0.0001

[a]Treatment results are presented in Figure 2 of the article. p<0.05 was considered statistically significant. Terms that were significant predictors of abstinence for weeks 9–12 and/or 9–24 are shown. Non-significant candidate terms included: treatment by medical history cohort interaction, gender, alcohol and/or substance dependence/use/abuse comorbidity, alcohol dependence or abuse comorbidity, substance dependence or use comorbidity, C-SSRS lifetime suicidality behavior and/or ideation, C-SSRS lifetime suicidality behavior, C-SSRS lifetime suicidality ideation, prior use of varenicline, prior use of bupropion, lives with a smoker, age started smoking, HADS Anxiety at baseline, HADS Depression at baseline.

BMI: body mass index; CA: continuous abstinence; CI: confidence interval; C-SSRS: Columbia-Suicide Severity Rating Scale; FTCD: Fagerström Test for Cigarette Dependence; HADS: Hospital Anxiety and Depression Scale; NRT: nicotine replacement therapy; NS: not significant; OR: odds ratio.

Table S3 Stepwise logistic modeling: analysis of varenicline (or bupropion or NRT) versus placebo across smoking-related-disease subcohorts in all randomized EAGLES participants[a]

	Asthma		COPD		Diabetes		CVD		No smoking-related diseases[b]	
	**OR**	**95% CI**	**OR**	**95% CI**	**OR**	**95% CI**	**OR**	**95% CI**	**OR**	**95% CI**
**Varenicline vs. placebo**										
CA9-12	1.51	0.81–2.83	4.42	2.01–9.70	8.09	3.49–18.76	2.31	0.96–5.59	3.74	3.10–4.50
CA9-24	1.86	0.83–4.16	3.87	1.42–10.58	6.87	2.48–19.05	1.28	0.46–3.57	2.83	2.29–3.48
**Bupropion vs. placebo**										
CA9-12	1.09	0.55–2.19	2.29	1.00–5.24	3.79	1.61–8.93	2.00	0.82–4.85	2.12	1.75–2.58
CA9-24	1.73	0.74–4.02	1.80	0.60–5.45	3.94	1.39–11.17	1.09	0.38–3.12	1.93	1.55–2.40
**NRT vs. placebo**										
CA9-12	0.79	0.39–1.62	2.33	1.03–5.28	4.33	1.82–10.27	1.98	0.82–4.81	2.24	1.84–2.71
CA9-24	0.82	0.31–2.13	2.47	0.87–7.03	5.01	1.77–14.17	0.72	0.23–2.22	1.85	1.48–2.30

Model terms included treatment, psychiatric cohort (psychiatric vs. non-psychiatric), and region (non-USA vs. USA).

[a]Participants may appear in multiple smoking-related-disease subcohorts.

[b]Participants without asthma, COPD, diabetes, or CVD.

CA: continuous abstinence; CI: confidence interval; COPD: chronic obstructive pulmonary disease; CVD: cardiovascular disease; OR: odds ratio.

Table S4 Weight and change from baseline weight by treatment and cohort in all treated EAGLES participants

	All disease subcohorts[a]					No smoking-related diseases[b]				
	**Varenicline**	**Bupropion**	**NRT**	**Placebo**	**All**	**Varenicline**	**Bupropion**	**NRT**	**Placebo**	**All**
Baseline weight										
n	338	332	334	351	1355	1496	1487	1498	1461	5942
Mean (SD), kg	85.5 (21.7)	84.7 (22.5)	83.2 (20.2)	84.2 (20.5)	84.4 (21.2)	80.0 (20.0)	80.1 (20.0)	80.1 (19.5)	80.3 (20.0)	80.1 (19.9)
Weight change from baseline: week 12										
n	278	289	284	294	1145	1291	1266	1265	1246	5068
Mean (SD), kg	1.2 (2.9)	0.6 (2.8)	1.3 (2.9)	0.5 (3.0)	0.9 (2.9)	1.3 (2.8)	0.7 (2.7)	1.1 (2.7)	0.8 (2.6)	1.0 (2.7)
Weight change from baseline: week 24										
n	251	262	248	272	1033	1169	1128	1122	1097	4516
Mean (SD), kg	1.8 (4.3)	1.2 (4.1)	1.3 (3.5)	0.7 (3.8)	1.2 (3.9)	1.8 (3.8)	1.3 (3.7)	1.3 (3.6)	0.9 (3.6)	1.4 (3.7)

[a]Participants may appear in multiple smoking-related-disease subcohorts.

[b]Participants without asthma, COPD, diabetes, or CVD.

COPD: chronic obstructive pulmonary disease; CVD: cardiovascular disease; NRT: nicotine replacement therapy; SD: standard deviation.

Table S5 Common adverse events[a] by treatment and cohort for all treated EAGLES participants

	All disease subcohorts[a], n (%)					No smoking-related diseases[b], n (%)				
**MedDRA PT[c]**	**Varenicline (n=340)**	**Bupropion (n=333)**	**NRT (n=336)**	**Placebo (n=354)**	**All (n=1363)**	**Varenicline (n=1505)**	**Bupropion (n=1494)**	**NRT (n=1503)**	**Placebo (n=1468)**	**All (n=5970)**
Abnormal dreams	37 (10.9)	25 (7.5)	43 (12.8)	15 (4.2)	120 (8.8)	137 (9.1)	78 (5.2)	173 (11.5)	70 (4.8)	458 (7.7)
Agitation	11 (3.2)	18 (5.4)	12 (3.6)	7 (2.0)	48 (3.5)	53 (3.5)	44 (2.9)	46 (3.1)	46 (3.1)	189 (3.2)
Anxiety	24 (7.1)	29 (8.7)	28 (8.3)	21 (5.9)	102 (7.5)	88 (5.8)	11 (7.4)	91 (6.1)	85 (5.8)	375 (6.3)
Application site pruritus	6 (1.8)	3 (0.9)	15 (4.5)	4 (1.1)	28 (2.1)	14 (0.9)	9 (0.6)	80 (5.3)	11 (0.7)	114 (1.9)
Bronchitis	9 (2.6)	10 (3.0)	17 (5.1)	14 (4.0)	50 (3.7)	17 (1.1)	18 (1.2)	17 (1.1)	22 (1.5)	74 (1.2)
Constipation	18 (5.3)	20 (6.0)	9 (2.7)	6 (1.7)	53 (3.9)	65 (4.3)	48 (3.2)	34 (2.3)	28 (1.9)	175 (2.9)
Diarrhea	18 (5.3)	16 (4.8)	14 (4.2)	14 (4.0)	62 (4.5)	51 (3.4)	32 (2.1)	57 (3.8)	37 (2.5)	177 (3.0)
Dizziness	16 (4.7)	16 (4.8)	23 (6.8)	16 (4.5)	71 (5.2)	58 (3.9)	77 (5.2)	55 (3.7)	40 (2.7)	230 (3.9)
Dry mouth	13 (3.8)	34 (10.2)	12 (3.6)	10 (2.8)	69 (5.1)	51 (3.4)	98 (6.6)	43 (2.9)	45 (3.1)	237 (4.0)
Dysgeusia	13 (3.8)	17 (5.1)	7 (2.1)	11 (3.1)	48 (3.5)	35 (2.3)	62 (4.1)	27 (1.8)	22 (1.5)	146 (2.4)
Fatigue	26 (7.6)	12 (3.6)	20 (6.0)	18 (5.1)	76 (5.6)	78 (5.2)	34 (2.3)	47 (3.1)	51 (3.5)	210 (3.5)
Headache	46 (13.5)	27 (8.1)	44 (13.1)	37 (10.5)	154 (11.3)	176 (11.7)	137 (9.2)	175 (11.6)	147 (10.0)	635 (10.6)
Insomnia	33 (9.7)	41 (12.3)	33 (9.8)	29 (8.2)	136 (10.0)	134 (8.9)	181 (12.1)	142 (9.4)	99 (6.7)	556 (9.3)
Irritability	13 (3.8)	18 (5.4)	16 (4.8)	12 (3.4)	59 (4.3)	59 (3.9)	42 (2.8)	81 (5.4)	78 (5.3)	260 (4.4)
Nasopharyngitis	34 (10.0)	23 (6.9)	25 (7.4)	30 (8.5)	112 (8.2)	125 (8.3)	120 (8.0)	91 (6.1)	95 (6.5)	431 (7.2)
Nausea	83 (24.4)	36 (10.8)	37 (11.0)	30 (8.5)	186 (13.6)	379 (25.2)	142 (9.5)	146 (9.7)	94 (6.4)	761 (12.7)
Sleep disorder	17 (5.0)	9 (2.7)	7 (2.1)	9 (2.5)	42 (3.1)	43 (2.9)	55 (3.7)	31 (2.1)	29 (2.0)	158 (2.6)
Upper respiratory tract infection	27 (7.9)	22 (6.6)	21 (6.3)	28 (7.9)	98 (7.2)	66 (4.4)	68 (4.6)	63 (4.2)	69 (4.7)	266 (4.5)

[a]Participants may appear in multiple smoking-related-disease subcohorts.

[b]Participants without asthma, COPD, diabetes, or CVD.

[c]Reported in ≥5% of participants in any treatment by cohort group.

MedDRA: Medical Dictionary for Regulatory Activities; NRT: nicotine replacement therapy; PT: preferred term.

Figure S1 Participant flow diagram

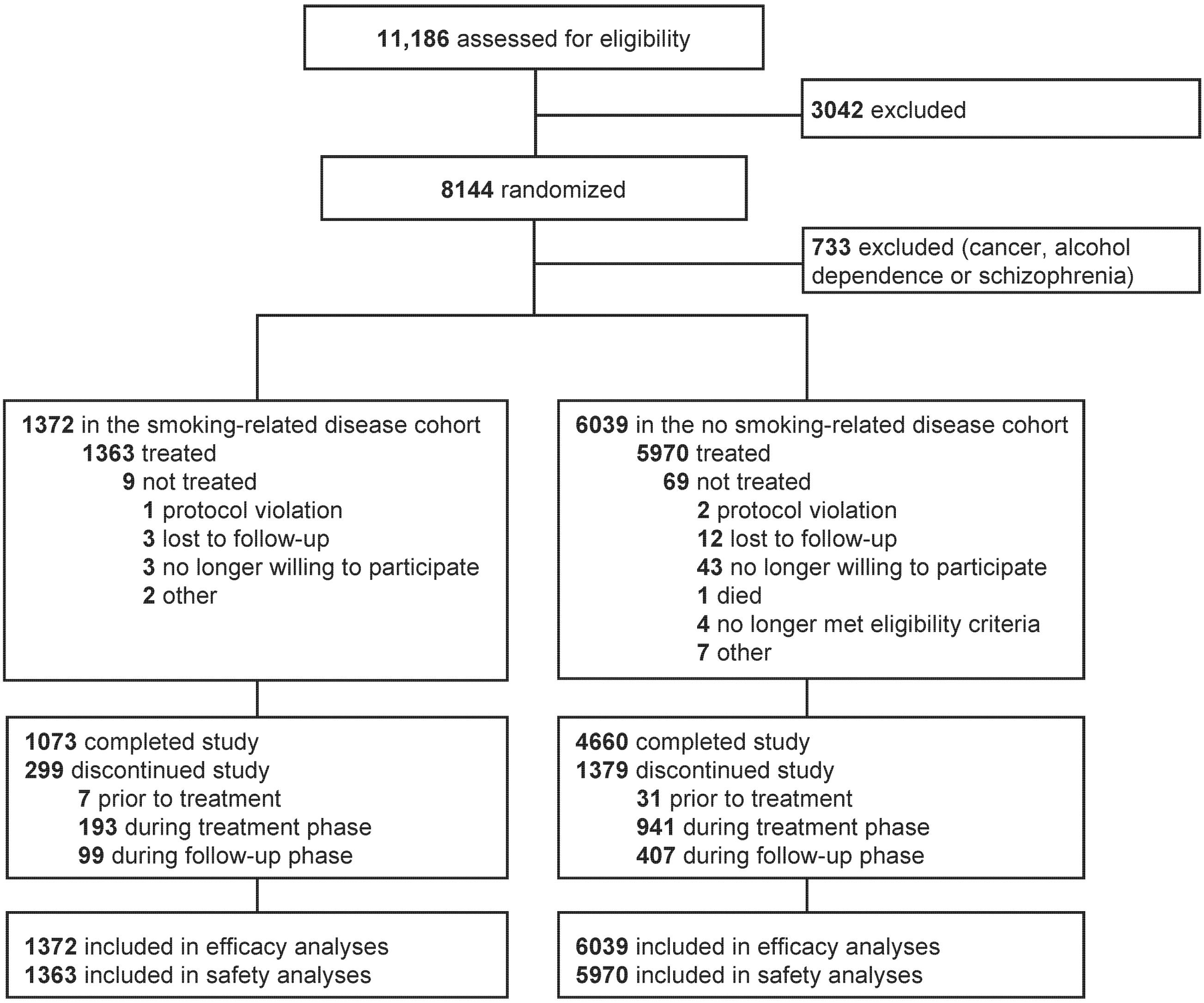

Figure S2 Comparison of continuous abstinence rates at weeks 9–12 in randomized trials of smokers with and without smoking-associated diseases

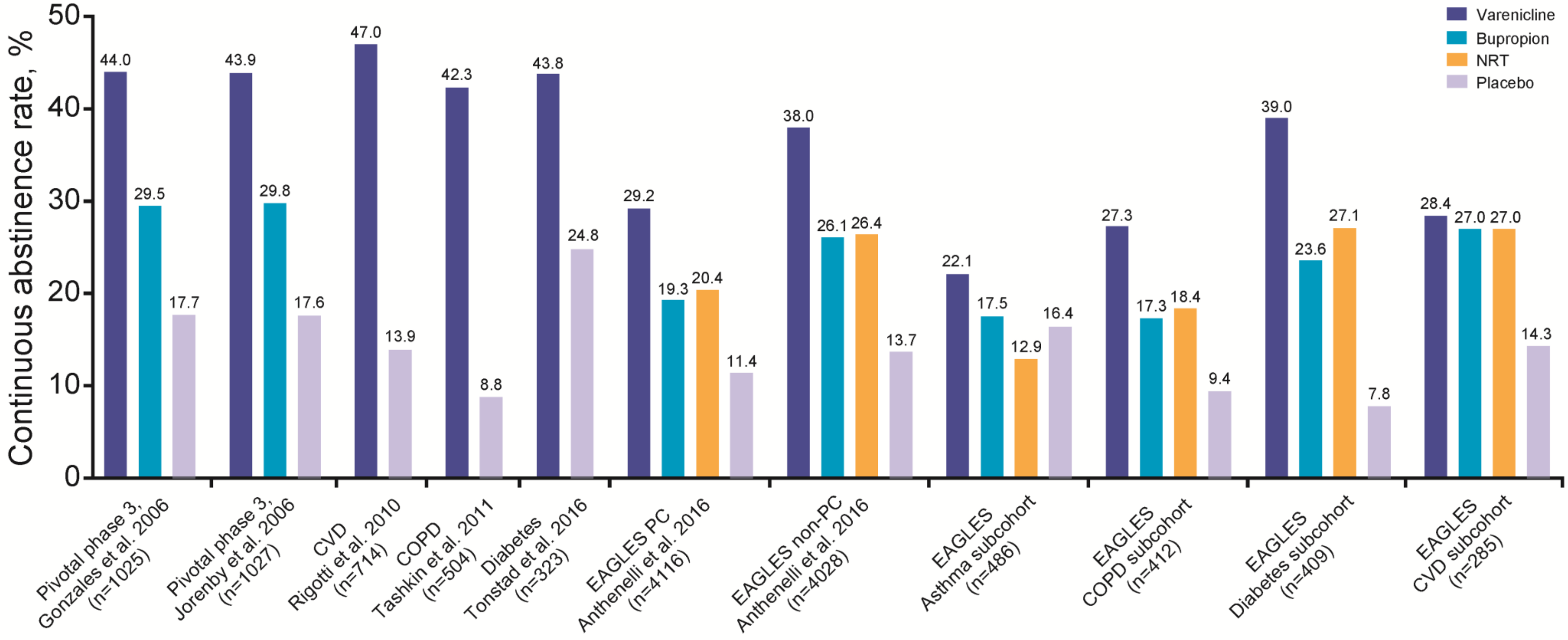

COPD: chronic obstructive pulmonary disease; CVD: cardiovascular disease; EAGLES: Evaluating Adverse Events in a Global Smoking Cessation Study; NRT: nicotine replacement therapy; PC: psychiatric cohort

Figure S3 Comparison of continuous abstinence rates at weeks 9–24 in randomized trials of smokers with and without smoking-associated diseases

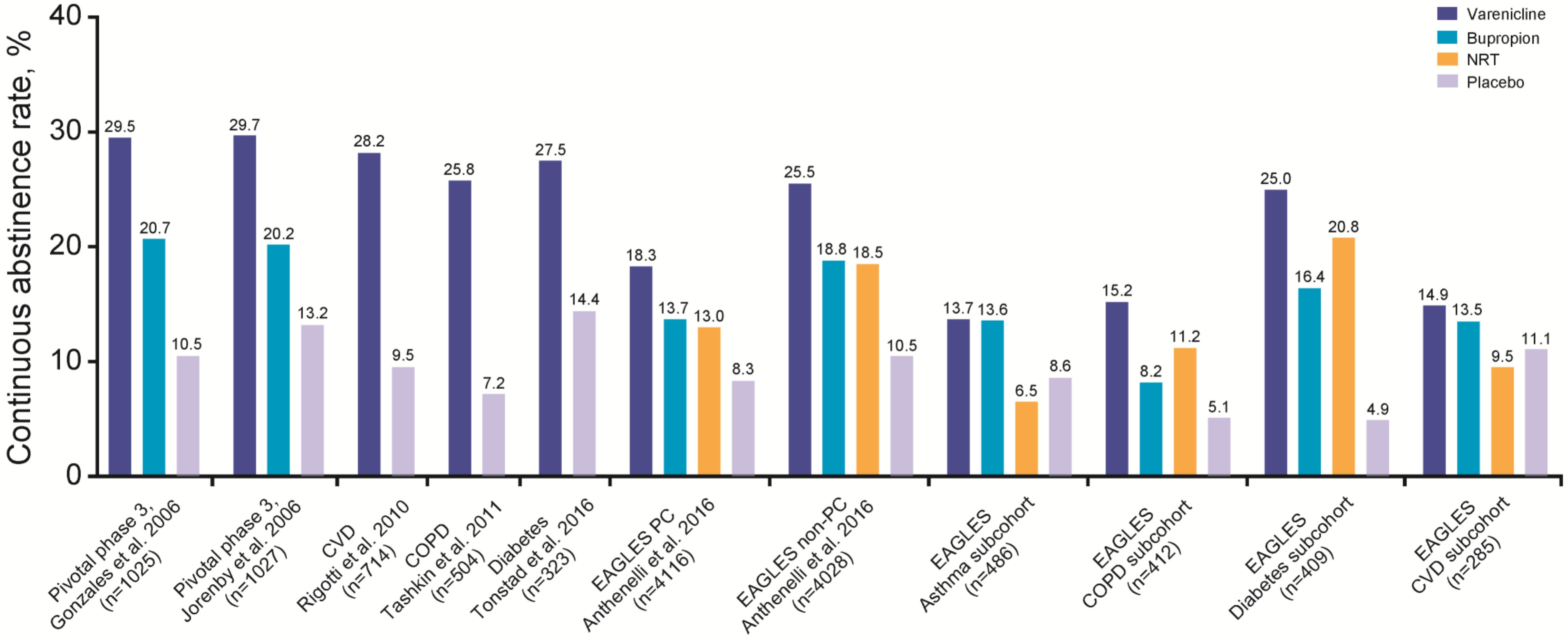

COPD: chronic obstructive pulmonary disease; CVD: cardiovascular disease; EAGLES: Evaluating Adverse Events in a Global Smoking Cessation Study; NRT: nicotine replacement therapy; PC: psychiatric cohort

© 2022 Tønnesen P. et al.
